# Defining early recurrence in patients with resected primary colorectal carcinoma and its respective risk factors

**DOI:** 10.1007/s00384-021-03844-7

**Published:** 2021-01-15

**Authors:** Felix Wiesmueller, Rolf Schuetz, Melanie Langheinrich, Maximilian Brunner, Georg F. Weber, Robert Grützmann, Susanne Merkel, Christian Krautz

**Affiliations:** Department of Surgery, University Hospital Erlangen, Friedrich-Alexander-University of Erlangen-Nuremberg (FAU), Krankenhausstr. 12, 91054 Erlangen, Germany

**Keywords:** Colorectal cancer, Colorectal carcinoma, Cancer recurrence, Recurrence group, Follow-up, Hemicolectomy, Low anterior resection

## Abstract

**Purpose:**

There is no evidence-based definition of early recurrence following resection of colorectal cancer. The purpose of this study is to define a point that discriminates between early and late recurrence in patients who have undergone colorectal cancer resection with curative intent and to analyze associated risk factors.

**Methods:**

A retrospective single-center cohort study was performed at a university hospital recognized as a comprehensive cancer center, specializing in colorectal cancer surgery. Patient data were retrieved from a prospectively maintained institutional database. Included patients underwent resection for primary, non-metastatic colorectal carcinomas with curative intent between 1995 and 2010. Aims of the study were (1) to define the optimal cut-off point of recurrence-free survival based on overall survival using a minimum *p* value approach and (2) to identify patterns of initial recurrence and putative risk factors for early recurrence using regression models.

**Results:**

Recurrence was diagnosed in 412 of 1893 patients. Statistical analysis suggested that a recurrence-free survival of 16 months could be used to distinguish between early and late recurrence based on overall survival (*p* < 0.001). Independent risk factors for early recurrence included advanced pT categories (pT3,4/ypT3,4) and positive lymph node status (pN+/ypN+). Early recurrence was independent of site of recurrence and was associated with worse prognosis.

**Conclusions:**

Recurrence of colorectal carcinoma within 16 months after primary treatment should be labeled as “early.” Tumor categories pT3,4/ypT3,4 and positive lymph node status pN+/ypN+ are predictive of early recurrence.

## Introduction

Colorectal cancer (CRC) has the third highest tumor incidence in both men and women, making it one of the most common malignancies. A combined mortality rate of 14.2% renders this entity the second most common cause of cancer-related deaths [1]. Therapy depends on tumor stage and typically consists of surgical resection, which may be combined with other treatment modalities, such as chemotherapy or radiation. During post-treatment surveillance, patients are monitored with regular physical examination, measurement of tumor markers, imaging studies, and endoscopies [2]. Patients with recurrence may benefit from additional therapy [3]. Major medical association guidelines categorize follow-up schedules in an early period with more frequent examinations and a late period with less frequent examinations. However, evidence-based distinction of recurrence groups in CRC is currently lacking [2].

Several studies have used a minimum *p* value approach to determine cutoff points for defining recurrence groups in other tumor entities [4–6]. We hypothesized that patients with CRC recurrence following primary resection can be dichotomized into an early and a late recurrence group based on their respective survival. To achieve this, we intended to use a minimum *p* value approach. Additionally, we intended to examine risk factors that might be associated with the resulting recurrence groups.

## Material and methods

### Patient data

This retrospective study was performed at the Department of Surgery, University Hospital Erlangen, Friedrich-Alexander-University of Erlangen-Nuremberg (FAU), Germany. It was approved by the local Ethics Commission (submission ID: 414_18 Bc). All patient information was retrieved from the Erlangen Registry for Colorectal Carcinomas (ERCRC), an institutional database that is maintained in a prospective manner. Patient data included epidemiologic parameters, clinical findings, treatment history, imaging results, histologic results, and follow-up data collected either at the university hospital, or from family physicians of patients via mailed questionnaires for at least 5 years. Follow-up was performed at 3-month intervals for the first 2 years, and at 6-month intervals thereafter. In 2004, follow-up intervals were changed to 6-month intervals for the first 2 years and then yearly for the following 3 years, in accordance with the first edition of the German Evidence-based Guideline for Colorectal Cancer [7]. After 5 years of tumor-free follow-up, ERCRC contacted patients at their local registration by telephone to check for their status (living or deceased).

### Aims and criteria

The aim of the study was to analyze a cohort of patients with local and/or distant recurrence following surgical resection of primary, not metastasized carcinoma of the colon or rectum between 1995 and 2010. Primary endpoints were recurrence-free survival (RFS) and overall survival (OS). Secondary aims were (1) to assess demographic, clinicopathologic and treatment characteristics of the patient collective and (2) to perform logistic regression on these characteristics in order to identify possible risk factors for early recurrence.

Inclusion criteria were solitary invasive colorectal carcinoma (tumor stages T1 and above), treatment by hemicolectomy with complete mesocolic excision (CME) [8], extended hemicolectomy with CME, low anterior resection, intersphincteric resection or abdominoperineal excision with total mesorectal excision (TME) [9] at the Department of Surgery of the University Hospital Erlangen, treatment between 1995 and 2010, macroscopic curative resection (R0, R1), no other previous or synchronous malignancies, no history of familial adenomatous polyposis, ulcerative colitis or Crohn’s disease, no distant metastases, no watch and wait strategy with secondary resection. Exclusion criteria were surgery-related mortality, death within 90 days of operation, unknown tumor status, < 84 months of follow-up without recurrence or death.

### Clinical and pathological variables

The primary tumor was categorized as rectal if located ≤16 cm from the anal verge when measured with a rigid rectosigmoidoscope. Primary colorectal tumors located proximal to this limit were categorized as colonic tumors. Patients were assorted to pT1,2/ypT0,1,2, pT3,4/ypT3,4, pN0/ypN0, or pN+/ypN+ groups according to their respective TNM stage. The TNM stage was determined as specified by the UICC/AJCC Cancer Staging Manual, 8th Edition [10]. Treatment was divided into either “surgery only” or “multimodal,” if patients received any form of additional therapy pre- or postoperatively, e.g., chemotherapy. Surgery was considered elective if patients were regularly admitted for a planned procedure, contrary to emergent procedures, defined as the need for urgent surgery within 48 h of admission [11]. The American Society of Anesthesiologists (ASA) scoring system was applied preoperatively to assess the overall fitness of patients and documented in the patient chart. These retrieved ASA scores, if available, were grouped as either ASA 1–2 or ASA 3–4. CEA concentrations of ≥5 ng/ml were considered pathologically elevated. Resection margins were rated as either R0 (free resection margins) or R1 (microscopically positive margins) after histopathologic workup. The histomorphologic diagnosis of this workup was recorded as either adenocarcinoma or mucinous/signet ring cell carcinoma. Postoperative complications were defined as a deviation from the normal postoperative course requiring treatment.

### Recurrences

Our institutional follow-up strategy adhered to national guidelines [12]. Symptomatic as well as asymptomatic recurrences were recorded. The first occurrence/event of recurrence was analyzed. Recurrent disease was classified as “local,” “distant,” (metastases) or “both” according to restaging results from follow-up imaging. Distant sites of recurrence were further stratified into “liver metastases,” “pulmonary metastases,” “other sites,” or “multiple sites of metastases.” The study collective was further assessed according to gender (male / female), age at time of diagnosis (< 65 years / ≥ 65 years of age), and date of procedure (1995–2003 / 2004–2010).

### Statistical analysis

The overall survival (OS) was calculated from the start of primary treatment to either death or date of last follow-up. The recurrence-free survival (RFS) was defined as the period between procedure and date of first locoregional and/or distant recurrence. A minimum *p* value approach was used to identify patterns of recurrence assuming that there are two groups of recurrence defined by a single cut-off point in time. In order to define the optimal cut-off point, a piecewise regression with log-rank tests was performed to compare OS of early and late recurrence groups at different thresholds. These thresholds were formulated in progressing steps of 3 months, starting at 6 months until a maximum of 60 months after the procedure. Median OS was calculated for both putative recurrence groups at each cut-off value. The lowest resulting *p* value was chosen as an optimal cut-off point. Potential risk factors for early colorectal recurrence were assessed by logistic regression. Cox’s proportional hazard model was used to assess risk factors for OS. Variables that showed an association with a *p* value of <0.05 in univariate analysis were included as covariates in a multivariate regression model. Results of both analyses were displayed as odds ratios (OR) or hazard ratios (HR) with their corresponding 95% confidence interval (CI). Statistical significance was defined as *p* < 0.05. We used the STROBE cohort checklist when writing our report [13]. The Sankey diagram was created with Tableau Desktop 2019.2 (Tableau Software, Seattle, WA). The minimum *p* value graph was plotted using Microsoft Excel 2011 (Microsoft Corp., Redmond, WA). All other graphs and statistical analyses were prepared with SPSS 21.0 software (IBM Corp., Armonk, NY).

## Results

### Patient cohort

A total of 1893 patients were included in the study. Two hundred sixty-two patients had rectal cancer, 150 patients had colonic cancer. Twenty (1.1%) patients were excluded due to unknown tumor status. Four hundred twelve (21.8%) patients developed recurrence. These patients were further analyzed. The median OS for all patients with recurrence was 58 months (95% CI 14–198 months). The median RFS of the cohort was 21 months (95% CI 5–83 months). At the time of analysis 348 patients (84.5%) had died. Further clinicopathologic characteristics are summarized in Table 1.

**Table 1:** Patient and tumor characteristics

Variable	Total number of patients (*n* = 1873)	Percentage (%)
Location of primary tumor in patients without recurrence		
Rectum	741	39.6
Left Colon	422	22.5
Right Colon	298	15.9
	Number of Patients with recurrence (*n* = 412)	Percentage (%)
Gender		
Male	271	65.8
Female	141	34.2
Age at time of diagnosis (years)		
< 65	211	51.2
≥ 65	201	48.8
Location of primary tumor		
Rectum	262	63.6
Left Colon	95	23.1
Right Colon	55	13.3
pT category*		
pT1,2/ypT0,1,2	97	23.5
pT3,4/ypT3,4	315	76.5
pN category*		
pN0/ypN0	195	47.3
pN+/ypN+	217	52.7
Time of surgery		
1995–2003	257	62.4
2004–2010	155	37.6
Treatment		
Surgery only	181	43.9
Multimodal treatment	231	56.1
Type of admission		
Elective surgery	382	92.7
Emergency surgery	30	7.3
ASA score^†^		
1–2	309	80.0
3–4	77	20.0
CEA concentration (ng/ml)^††^		
< 5	235	57.0
≥ 5	98	23.8
Resection margin		
R0	401	97.3
R1	11	2.7
Histomorphology		
Adenocarcinoma	372	90.3
Mucinous carcinoma /signet-ring cell carcinoma	40	9.7
Postoperative complications		
No complications	301	73.1
Any complications	111	26.9
Site of recurrence		
Locoregional	43	10.4
Distant	292	70.9
Local and distant	77	18.7
Liver metastases	120	29.1
Lung metastases	68	16.5
Other metastasis sites	39	9.5
Multiple metastasis sites	57	13.8

*American Joint Committee on Cancer (AJCC) and Union for International Cancer Control (UICC) staging system [10]

^†^Three hundred eighty-six patients had preoperative American Society of Anesthesiologists (ASA) scores available for analysis

^††^Three hundred thirty-three patients had preoperative CEA levels available for analysis

### Early and late recurrence

The optimal cut-off point of RFS to differentiate between an early and a late recurrence group based on the overall prognosis was found to be at 16 months after primary treatment. The early recurrence group consisted of 157 patients, while the late recurrence group included 255 patients. Median OS of the early recurrence group was 33 months, while the late recurrence group had a median OS of 77 months (*p* < 0.001). Five-year post-recurrence survival of the early recurrence group was 18.5% (95% CI 12.4–24.6) vs. 31.6% (25.7–37.5) of the late recurrence group (*p* = 0.004).

**Fig. 1:**
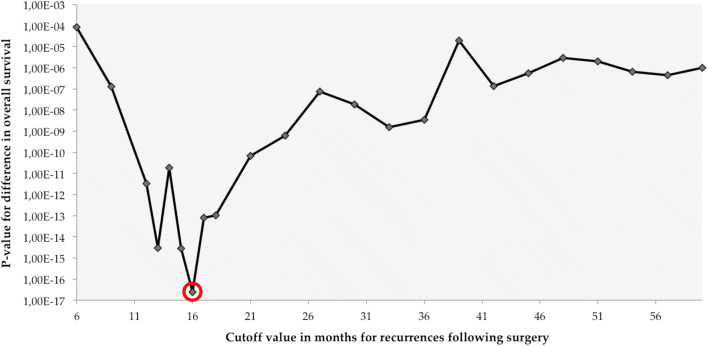
Optimal cut-off point displayed by the course of different cutoff points with corresponding *p* values plotted on a logarithmic scale. The optimal cutoff threshold at 16 months (circled in red) distinguishes between an early and a late recurrence group in terms of the overall survival

Results of the minimum *p* value approach are shown in Table 2. Additionally, a graph was plotted to demonstrate the trend of *p* values along the progressing putative thresholds with a nadir at 16 months (Fig. 1).

**Table 2:** Cut-off thresholds for determining recurrence groups based on overall survival

		Potential early recurrence group		Potential late recurrence group	
Evaluated cutoff point (months)	*P* value	n	Median OS (months)	n	Median OS (months)
< 6	0.000085	32	21	380	62
< 9	1.288 × 10^−7^	67	25	345	65
< 12	3.35 × 10^−12^	102	30	310	69
< 13	2.908 × 10^−15^	117	30	295	72
< 14	1.862 × 10^−14^	131	32	281	73
< 15	2.818 × 10^−15^	140	32	272	75
*< 16*	*2.467 × 10*^*−17*^	*157*	*33*	*255*	*77*
< 17	8.119 × 10^−14^	170	34	242	77
< 18	1.03 × 10^−13^	178	34	234	77
< 21	6.931 × 10^−11^	204	36	208	78
< 24	6.234 × 10^−10^	223	38	189	80
< 27	7.551 × 10^−8^	250	39	162	83
< 30	1.842 × 10^−8^	264	40	148	86
< 33	1.556 × 10^−9^	283	40	129	92
< 36	3.553 × 10^−9^	301	43	111	101
< 39	1.956 × 10^−8^	313	46	99	102
< 42	1.368 × 10^−7^	327	48	85	104
< 45	5.616 × 10^−7^	331	49	81	104
< 48	0.000003	336	49	76	103
< 51	0.000002	347	49	65	106
< 54	6.474 × 10^−7^	360	52	52	110
< 57	4.594 × 10^−7^	361	52	51	110
< 60	0.000001	364	52	48	117

Optimal cut-off point with lowest *p* value is marked in italics; *n* indicates the number of patients in each potential recurrence group; *OS*, overall survival

### Recurrence patterns

**Fig. 2:**
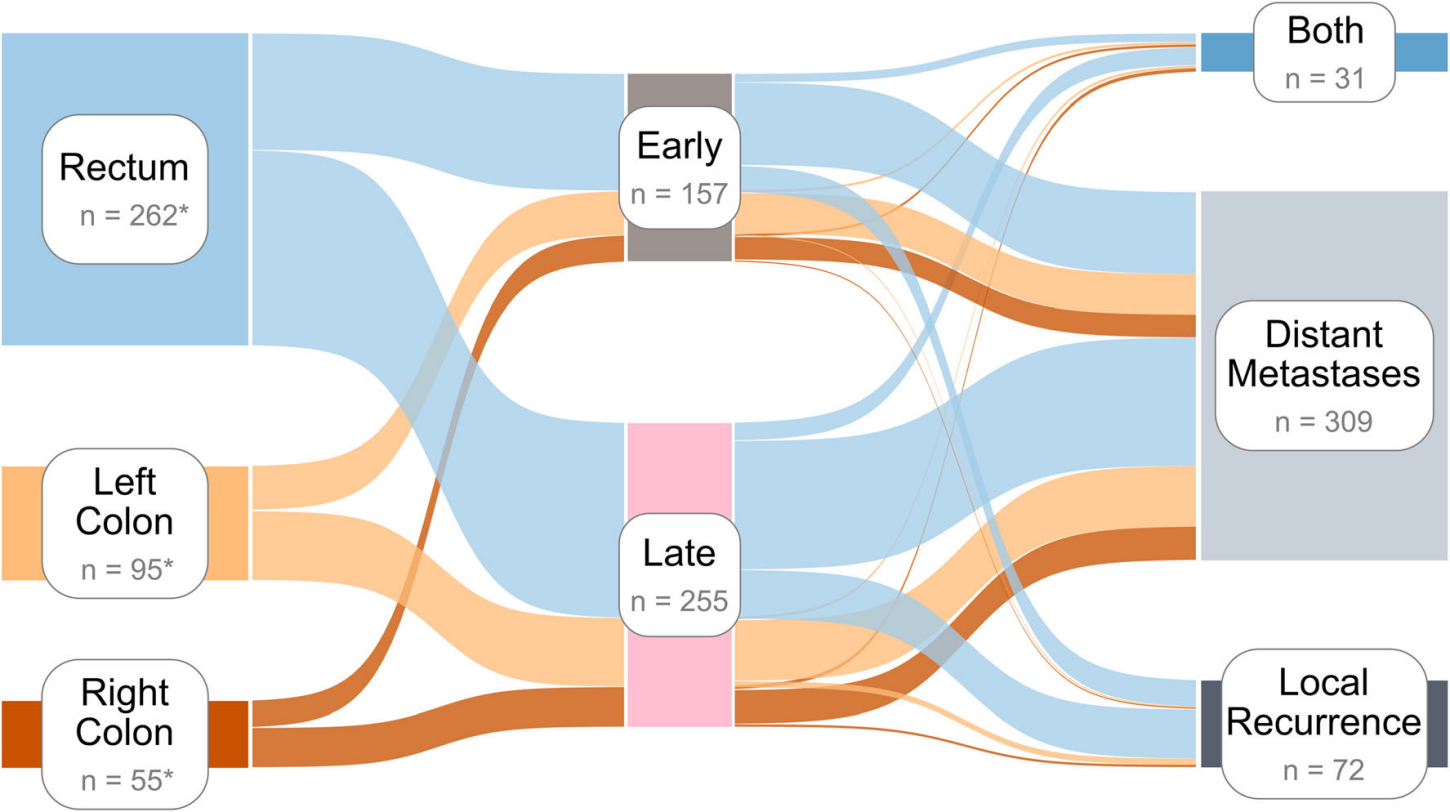
Sankey diagram of colorectal recurrences illustrating the proportions of recurrent colorectal tumors (rectum, left colon, right colon) that are diagnosed within (early) or after (late) 16 months following primary treatment as well as their corresponding sites of recurrence (Local Recurrence, Distant Metastases, Both). All primary tumors appear to be evenly distributed among early and late recurrence groups. Colonic tumors contribute more to distant metastases than to local or both (local + distant) recurrences. N indicates the number of respective cases; * total number of treated patients: rectum = 1003, left colon = 517, right colon = 353

**Fig. 3:**
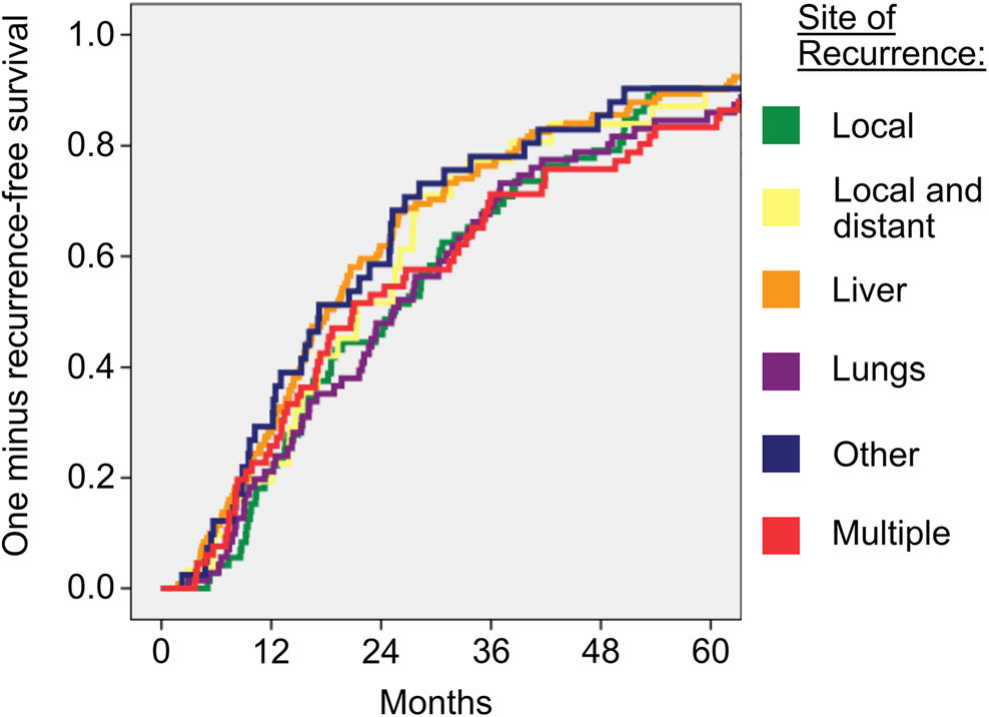
Kaplan-Meier estimator for comparison of site of recurrence and recurrence-free survival. There is no specific site that is favorable of early recurrence

**Fig. 4:**
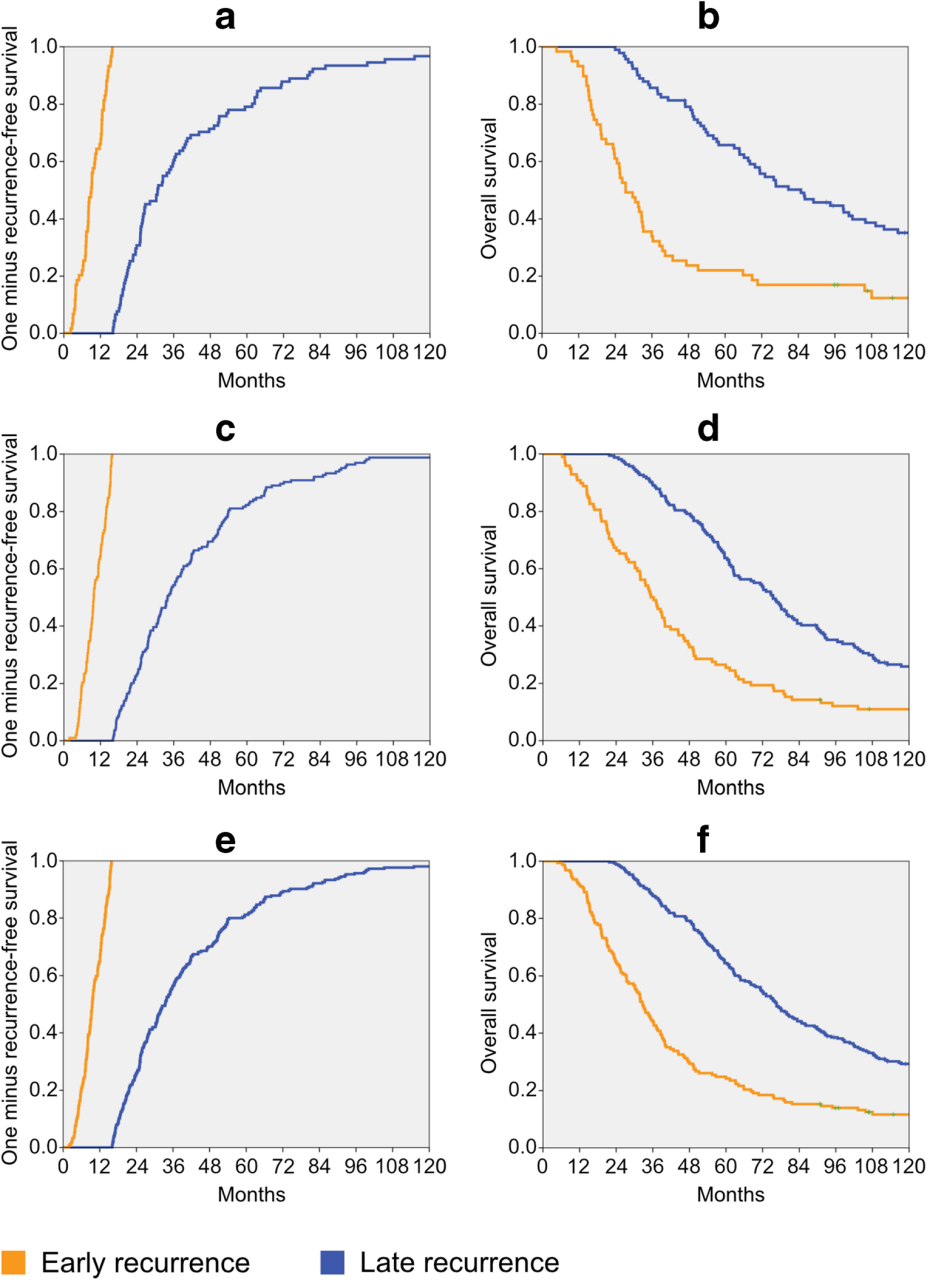
Kaplan-Meier curves of early and late recurrences for recurrence-free survival (**a**, **c**, **e**) and overall survival (**b**, **d**, **f**) in colonic (**a**, **b**), rectal (**c**, **d**), and both (**e**, **f**) carcinomas

Most recurrences arise from rectal carcinomas followed by primary tumors of the left and right colon (Fig. 2). This composition of recurrences is distributed similarly among early and late recurrence groups. Colonic carcinomas are most likely to recur as distant metastases, and less likely to recur as local or simultaneous local and distant recurrences. Recurrence-free survival was not influenced by the location of recurrent tumors (Fig. 3). Overall survival and recurrence-free survival were similar between location of primary tumors when dividing into early and late recurrence groups (Fig. 4).

### Risk factor assessment

Univariate analysis showed that advanced pT category (pT3,4/ypT3,4: OR 2.1, 95% CI 1.3–3.4, *p* = 0.005), positive lymph node status (pN+/ypN+: OR 2.4, 95% CI 1.6–3.6, *p* < 0.001), and positive resection margins (R1: OR 4.5, 95% CI 1.2–17.3, *p* = 0.028) were significantly associated with early recurrence (< 16 months) (Table 3). On multivariate analysis, advanced pT category (OR 1.8, 95% CI 1.1–3.0, *p* = 0.029) and positive lymph node status (OR 2.2, 95% CI 1.4–3.3, *p* < 0.001) proved to be independent risk factors (Table 3).

**Table 3:** Logistic regression on risk of early recurrence (< 16 months cutoff), 157 of 412 patients total

		Univariate analysis			Multivariate analysis		
Risk factors	*n*	Odds ratio	95% CI	*P* value	Odds ratio	95% CI	*P* value
Gender							
Male	271	1.0	0.9–2.0	0.181			
Female	141	1.3					
Age at time of diagnosis (years)							
< 65	211	1.0					
≥ 65	201	1.1	0.7–1.6	0.625			
Location of primary tumor							
Colon	150	1.1					
Rectum	262	1.0	0.7–1.6	0.698			
pT category*							
pT1,2/ypT0,1,2	97	1.0			1.0		
*pT3,4/ypT3,4*	*315*	*2.1*	*1.3–3.4*	*0.005*	*1.8*	*1.1–3.0*	*0.029*
pN category*							
pN0/ypN0	195	1.0			1.0		
*pN+/ypN+*	*217*	*2.4*	*1.6–3.6*	*< 0.001*	*2.2*	*1.4–3.3*	*< 0.001*
Time of surgery							
1995–2003	257	1.0					
2004–2010	155	0.9	0.6–1.4	0.665			
Treatment							
Surgery only	181	1.0					
Multimodal treatment	231	1.5	1.0–2.2	0.067			
Type of admission							
Elective surgery	382	1.0					
Emergency surgery	30	1.7	0.8–3.6	0.167			
ASA score^†^							
1–2	309	1.0					
3–4	77	0.9	0.7–1.2	0.588			
CEA concentration (ng/ml)^††^							
< 5	235	1.0					
≥ 5	98	1.6	1.0–2.6	0.053			
Resection margin							
R0	401	1.0			1.0		
*R1*	*11*	*4.5*	*1.2–17.3*	*0.028*	*3.7*	*1.0–14.6*	*0.057*
Histomorphology							
Adenocarcinoma	372	1.0					
Mucinous carcinoma / signet-ring cell carcinoma	40	1.1	0.6–2.1	0.795			
Postoperative complications							
No complications	301	1.0					
Any complications	111	1.1	0.7–1.7	0.697			
Site of recurrence							
Locoregional	43	1.0					
Local and distant	77	1.3	0.6–2.7	0.562			
Liver metastases	120	1.4	0.7–2.9	0.335			
Pulmonary metastases	68	0.8	0.3–1.8	0.546			
Other sites of metastases	39	1.3	0.5–3.2	0.567			
Multiple sites of metastases	57	0.9	0.4–2.0	0.728			

Significant results are marked in italics; *n* indicates the number of patients; *CI*, confidence interval

*American Joint Committee on Cancer (AJCC) and Union for International Cancer Control (UICC) staging system [10]

^†^Three hundred eighty-six patients had preoperative American Society of Anesthesiologists (ASA) scores available for analysis

^††^Three hundred thirty-three patients had preoperative carcinoembryonic antigen (CEA) levels available for analysis

An additional Cox regression analysis of overall survival in all patients (Table 4) showed that R1 resection margins versus R0 margins (HR 2.0, 95% CI 1.0–3.8, *p* = 0.038) and locoregional plus distant metastases (HR 3.7, 95% CI 2.3–5.8, *p* < 0.001) or multiple sites of metastases (HR 2.7, 95% CI 1.8–3.9, *p* < 0.001) versus pulmonary metastases alone significantly shortened OS. Late recurrences were also associated with a significantly longer OS compared to early recurrences (HR 0.4, 95% CI 0.3–0.5, *p* < 0.001).

**Table 4:** Cox regression for overall survival from start of primary treatment, 412 patients total

Risk factors	*n*	Hazard ratio	95% CI	*P* value
pT category*				
pT1,2/ypT0,1,2	97	1.0		
pT3,4/ypT3,4	315	1.1	0.9–1.4	0.443
pN category*				
pN0/ypN0	195	1.0		
pN+/ypN+	217	1.2	1.0–1.5	0.069
Resection margin				
R0	401	1.0		
*R1*	*11*	*2.0*	*1.0–3.8*	*0.038*
Site of recurrence				
Pulmonary metastases	68	1.0		
Locoregional recurrence	43	1.3	0.9–2.0	0.154
*Locoregional + distant metastases*	*77*	*3.7*	*2.3–5.8*	*< 0.001*
Liver metastases	120	1.4	1.0–1.9	0.051
Other sites of metastases	39	1.3	0.9–2.0	0.193
*Multiple sites of metastases*	*57*	*2.7*	*1.8–3.9*	*< 0.001*
Time of recurrence				
Early recurrence (< 16 months)	157	1.0		
*Late recurrence (> 16 months)*	*255*	*0.4*	*0.3–0.5*	*< 0.001*

Significant results are marked in italics; *n* indicates the number of patients; *CI*, confidence interval

*American Joint Committee on Cancer (AJCC) and Union for International Cancer Control (UICC) staging system [10]

## Discussion and conclusion

The purpose of postoperative follow-up in cancer patients is to detect tumor recurrence, allowing for timely treatment, and, ultimately, improving survival. Unfortunately, there is little robust data about recurrences in CRC and no evidence-based definition for early recurrence after resection. Our study observed that the optimal cut-off value to differentiate between early and late recurrences, based on overall prognosis, is 16 months. Consequently, postoperative recurrence up to an interval of 16 months is linked to poor OS, as evident in the median OS of 33 months of early recurrences compared to a median OS of 77 months of late recurrences. Likewise, 5-year post-recurrence survival was lower in the early recurrence group (18.5%) than in the late recurrence group (31.6%).

Within the published literature, postoperative cut-off points that help differentiate between early and late recurrence have been chosen arbitrarily and vary from 1 to 5 years [14–19]. Our study observed that the optimal cut-off value to differentiate between early and late recurrences, using a minimum *p* value approach based on overall prognosis, is 16 months. Grooth et al. [4] recently used a minimum p value approach to define recurrence groups in pancreatic cancer. They underlined that for the minimum *p* value approach they consciously chose the post-recurrence survival (PRS) because OS times in the late recurrence group would potentially introduce bias due to a longer recurrence-free interval. As this bias may apply for diseases with short post-recurrence survival times, such as pancreatic cancer, it is unlikely in diseases with numerous treatment options for recurrent disease and longer post-recurrence survival times, such as colorectal cancer. In addition, RFS, OS, and PRS are not inversely related in CRC. Based on these considerations, we elected to use the difference in overall survival in our analyses to define early and late recurrence.

A recent meta-analysis by Zhao et al. [20] suggests that intense follow-up does improve OS compared to less intense follow-up care. Meester et al. [21] conducted a microsimulation model where they compared benefits and costs of different surveillance models of patients who had undergone removal of colorectal adenomas. Their model suggests that high-intensity surveillance as recommended in the US provides modest but clinically relevant benefits over low-intensity surveillance at acceptable cost. However, guidelines by the German Guideline Recommendation Group Arbeitsgemeinschaft der Wissenschaftlichen Medizinischen Fachgesellschaften e.V. (AWMF) [12], the National Comprehensive Cancer Network (NCCN) [22], the European Society for Medical Oncology (ESMO) [23, 24], and the American Society of Clinical Oncology (ASCO) [25] vary in their recommendations for the initial period of intensive follow-up. On average, they advocate an intensive surveillance using history taking, physical exam, CEA concentrations, CT scans and endoscopies for up to 3 years after resection and a less intense follow-up thereafter. The definition of potential early and late recurrence groups is of paramount importance to determine adequate risk-adjusted follow-up strategies that also account for economic feasibility [3]. The impact of timing of recurrence revealed in this study may help to improve survival in two ways. First, it allows for a refinement of follow-up strategies through a more precise definition of patients that are at risk of early recurrence. Second, it may aid in decision-making regarding the treatment of recurrence through better prognostic stratification, as late recurrence is associated with higher percentage of 5-year post-recurrence survival and longer OS. For example, instead of treating a singular liver lesion with resection only it might be treated with additional chemotherapy if diagnosed within 16 months after primary resection.

In accordance with the current literature [14, 16, 17, 19, 26], this study identified advanced depth of invasion (pT3,4/ypT3,4) and positive lymph node status (pN+/ypN+) as independent risk factors that are associated with early recurrence of CRC. R1 resection margins were discovered to be a risk factor for early recurrence by univariate analyses. However, statistical significance was not reached in multivariate analysis, likely due to the small number of patients with R1 resection (*p* = 0.057). Positive resection margins signify microscopically visible groups of tumor cells up to the resection margin. The remaining portion of malignant cells in the body could develop faster into a noticeable tumor bulk than single metastatic cells since tumor growth is exponential. From this mechanistic perspective, it makes sense to consider R1 margins as a risk factor for early recurrence. Future studies with larger patient cohorts might be able to clarify this issue.

CRC guidelines approve screening for increased serum CEA concentrations during follow-up until 3–5 years after resection of CRC [12, 22–25]. Nevertheless, there is some controversy regarding the value of CEA surveillance [2]. It seems to be a prognostic risk factor for systemic recurrence, especially of liver metastases. Yet, it has not been shown to be prognostic for locoregional recurrence and has an inverse correlation with tumor grade. Elevated levels are also seen in patients with chronic renal failure and smokers [27]. In this study, CEA was not identified as a risk factor. However, CEA values were missing in 79 (19.2%) patients.

For the refinement of follow-up strategies, it is essential to understand associations between the sites and the timing of recurrences as well as the respective prognosis. We observed that primary colonic and rectal carcinomas had similar RFS and OS curves with early recurrences having a worse prognosis (Fig. 4). Early and late recurrences were evenly distributed among colonic and rectal carcinomas. Metastatic spread of primary tumors seemed to be more or less evenly distributed among anatomic locations with colonic tumors having a tendency to contribute more to distant metastases (Fig. 2). Combined metastases (locoregional and distant) or multiple sites of metastases were associated with decreased OS as compared to pulmonary metastases alone. Yet, early recurrence and recurrence-free survival were not influenced by the location of recurrent tumors.

Follow-up for CRC with focus on particular organs, e.g., targeted liver ultrasound, does not seem reasonable for the detection of early recurrences. Instead, our data support an intensive, whole body follow-up for patients at risk of early recurrences for at least 16 months after primary therapy. The use of risk factors for early recurrence, such as tumor extent or lymph node status, may enable a more individual adaption of follow-up strategies. Yet, there is still the need to develop better methods for detection of tumor recurrence in order to improve detection rates and, possibly, patient survival. Emerging prognostic markers, such as circulating tumor DNA (ctDNA) [28, 29], microRNA (miRNA) [30, 31], and circulating tumor cells [32] may help to improve monitoring of CRC recurrence.

This study has several limitations. Generally, the minimum *p* value approach may increase Type I error [33] and it was assumed that there is only one but not several cut-off points. The retrospective nature of this single-center study and the limited size of the patient cohort may contribute to additional bias. The cohort featured patients from a broad time frame (1995–2010). Primary treatment and therapy of tumor recurrences underwent significant changes at our institution during this period of time: FOLFOX and FOLFIRI chemotherapy regimens were launched from 1995 to 2004, while novel biologic agents, e.g., vascular endothelial growth factor (VEGF) antibodies and epidermal growth factor receptor (EGFR) inhibitors were introduced during 2005 and 2010 [34]. Hence, we included different time periods of surgery into our multivariate regression models to acknowledge the confounding effect of time-related changes.

## Conclusion

After primary resection of CRC, patients with advanced pT category and/or positive lymph node status are at risk of early recurrence that may be defined as recurrence within 16 months. Early recurrence is associated with worse prognosis. Site of recurrence is not related to early recurrence. Having a clear threshold of postoperative survival that defines recurrence would allow better risk-adapted follow-up strategies in the future.

## Data Availability

Erlangen Registry for Colorectal Carcinomas (ERCRC).
